# A Machine Learning Approach to Estimate Hip and Knee Joint Loading Using a Mobile Phone-Embedded IMU

**DOI:** 10.3389/fbioe.2020.00320

**Published:** 2020-04-15

**Authors:** Arne De Brabandere, Jill Emmerzaal, Annick Timmermans, Ilse Jonkers, Benedicte Vanwanseele, Jesse Davis

**Affiliations:** ^1^Department of Computer Science, KU Leuven, Leuven, Belgium; ^2^Department of Movement Sciences, KU Leuven, Leuven, Belgium; ^3^Faculty of Rehabilitation Sciences, Hasselt University, Hasselt, Belgium

**Keywords:** machine learning, inertial measurement units, joint loading, patient monitoring, hip osteoarthrithis

## Abstract

Hip osteoarthritis patients exhibit changes in kinematics and kinetics that affect joint loading. Monitoring this load can provide valuable information to clinicians. For example, a patient's joint loading measured across different activities can be used to determine the amount of exercise that the patient needs to complete each day. Unfortunately, current methods for measuring joint loading require a lab environment which most clinicians do not have access to. This study explores employing machine learning to construct a model that can estimate joint loading based on sensor data obtained solely from a mobile phone. In order to learn such a model, we collected a dataset from 10 patients with hip osteoarthritis who performed multiple repetitions of nine different exercises. During each repetition, we simultaneously recorded 3D motion capture data, ground reaction force data, and the inertial measurement unit data from a mobile phone attached to the patient's hip. The 3D motion and ground reaction force data were used to compute the ground truth joint loading using musculoskeletal modeling. Our goal is to estimate the ground truth loading value using only the data captured by the sensors of the mobile phone. We propose a machine learning pipeline for learning such a model based on the recordings of a phone's accelerometer and gyroscope. When evaluated for an unseen patient, the proposed pipeline achieves a mean absolute error of 29% for the left hip and 36% for the right hip. While our approach is a step in the direction of using a minimal number of sensors to estimate joint loading outside the lab, developing a tool that is accurate enough to be applicable in a clinical context still remains an open challenge. It may be necessary to use sensors at more than one location in order to obtain better estimates.

## 1. Introduction

Hip osteoarthritis (OA) patients exhibit changes in kinematics and kinetics that affect the contact forces of the hip and knee joints during walking and daily activities. It is believed that these changes are important in the progression of OA (Felson, 2013) and that monitoring these changes during daily life could provide valuable information to clinicians. For example, a patient's joint loading measured across different exercises can serve as an indication for the number of exercise repetitions that the patient needs to complete when rehabilitating after hip arthroplasty surgery. Despite the importance of joint loading monitoring, it is difficult to systematically and widely measure joint loading in a clinical environment. First, acquiring these measurements requires a lab environment consisting of optoelectronic cameras and ground reaction force plates. The cost and space required for such a setup makes this impractical to install in a clinician's practice. Second, it would be infeasible to analyze a large number of patients in a lab since collecting and processing the data is a time-consuming task. Third, in order to calculate joint contact forces, one would need to use a musculoskeletal modeling workflow, which requires expert knowledge.

Because of these drawbacks, clinicians could greatly benefit from a mobile system that is able to provide accurate joint loading estimates based on cheaper sensors. Ideally, such a system would be based on inexpensive, wearable sensors that the patients can easily use at the clinician's practice or even at home. Inertial measurement unit (IMU) sensors and electromyography (EMG) sensors are ideal candidates for this purpose as they are relatively cheap and have been applied successfully in a wide range of human movement analysis tasks (Zhang et al., 2011; Camomilla et al., 2018; De Brabandere et al., 2018; Op De Beéck et al., 2018). Designing such a system requires collecting data in a lab setting where a subject performs the relevant exercises while simultaneously recording data from the cheap portable sensors and the expensive, standard lab sensors. This enables either hand-crafting a model or applying a data-driven approach such as machine learning to learn a model that relates the data produced by the portable sensors to the ground truth joint loading estimated from the lab equipment. These predictive models can then be deployed outside the lab as they can make predictions about a subject's joint loading solely based on the data captured by cheaper sensors.

Related studies have proposed different models for estimating joint loading from wearable sensors. de Vries et al. (2012) proposed a neural network model which estimates several loading variables for the shoulder joint based on kinematics and EMG data. The kinematics were measured using four IMU sensors. While the model can be used in an ambulatory setting, it still requires a relatively large number of sensors. Moreover, the EMG measurement is somewhat intrusive as it requires attaching 13 electrodes to the person's body. Other work by Wesseling et al. (2018) proposed a model for estimating hip and knee joint contact forces based on IMU kinematics and ground reaction force (GRF) data. They found that the IMU kinematics were sufficient to estimate the hip contact forces reliably, which enables using the model outside a lab. However, the knee contact force model required both the IMU and GRF data. Hence, this has the same drawbacks as the lab sensors for calculating joint contact forces as it is challenging to measure GRF data in the wild. Stetter et al. (2019) proposed a model for predicting knee joint loading using two IMU sensors, one on the upper leg and one on the lower leg. However, similar to de Vries et al. (2012) and Wesseling et al. (2018), they evaluated the model on data from healthy subjects only. Applying the same model to patients may not work due to altered movement patterns. Other studies considered similar problems, such as estimating the daily cumulative joint loading (Robbins et al., 2009) and ground reaction forces (Guo et al., 2017; Karatsidis et al., 2017; Wouda et al., 2018).

The goal of this paper is to predict the joint loading of the left and right hip and knee based on IMU data collected from a mobile phone. First, we collect data using three types of sensors simultaneously: a hip-mounted phone, optoelectronic motion capture cameras and ground reaction force plates. We use the latter two to calculate the ground truth joint loading using a musculoskeletal modeling workflow. Second, we employ machine learning to automatically construct a model that can predict the ground truth joint loading on the basis of the IMU data collected from the mobile phone. Our approach confers two advantages over prior work. First, by relying on a mobile phone it both builds off an omnipresent technology and minimizes the number of required sensors. Hence, clinicians and possibly even patients will not need to rely on expensive specialized equipment. Second, we focus on hip OA patients instead of healthy subjects. Since clinicians see patients with abnormal movement patterns, we train and evaluate the model using data collected from a representative patient group.

## 2. Methods

### 2.1. Subjects

For this study, 20 patients with unilateral end-stage hip osteoarthritis were recruited from a local hospital (Ziekenhuis Oost Limburg, Belgium). They were included based on the following criteria: aged between 55 and 75 years; unilateral hip osteoarthritis; awaiting joint replacement surgery; Body Mass Index ≤30kg·m^−2^; able to walk 10 m; no cortiosteroid injection 3 months prior to inclusion; no joint replacements and no other musculoskeletal or neurological disorders that would affect movement pattern. Participants provided written informed consent prior to the start of the measurements. Out of these 20 patients, we select only those for which the mobile phone measurements were recorded correctly throughout the whole protocol, which corresponds to a subset of 10 patients. The ethical committee of the academic hospital Leuven approved the study (reference no. s-59857).

### 2.2. Protocol

Each patient performed multiple repetitions of nine types of exercises. Table 1 shows the number of repetitions per exercise. The exercise types are defined as follows:

- **Walk**: level walking at a self-selected speed, one repetition corresponds to one stride;
- **Ascend stairs** and **descend stairs**: at a self selected speed, without hand-held support on a standardized 4-step staircase, one repetition corresponds to one stride;
- **Sit down** and **stand up**: the height of the chair was standardized to participants knee height;
- **Forward lunge** and **side lunge**: step length standardized at 70% leg length;
- **Stand on one leg** (approx. 2 s) and **squat on one leg**: hands fixed at the side.

**Table 1:** Number of exercise repetitions per subject.

**Subject ID**	**W**	**AS**	**DS**	**SD**	**SU**	**FL**	**SL**	**SOL**	**SQOL**
1	13	9	10	10	10	10	10	10	9
2	10	9	9	10	10	8	10	10	10
3	10	10	7	11	11	10	10	10	10
4	10	10	10	10	10	10	9	10	9
5	10	10	9	10	10	9	10	10	10
6	10	12	8	10	10	10	9	10	10
7	10	10	10	10	10	10	10	10	10
8	10	9	10	10	10	10	10	11	10
9	13	10	12	10	10	10	10	10	12
10	10	10	10	10	10	10	10	10	10

*W, walk; AS, ascend stairs; DS, descend staris; SD, sit down; SU, stand up; FL, forward lunge; SL, side lunge; SOL, stand on one leg; SQOL, squat on one leg*.

### 2.3. Joint Loading

We measure the patients' hip and knee contact force to define the ground truth joint loading that we aim to estimate. While contact forces can be measured directly using instrument prostheses, we instead use a combination of experimental data and musculoskeletal modeling (Fregly et al., 2012) since the direct method is an invasive procedure. Moreover, this method requires total joint replacement, which would limit the number of patients we can analyze. The remainder of this section describes the procedure for calculating the contact forces. Validation studies by Wesseling et al. (2016) and Zargham et al. (2019) have shown that this procedure results in accurate estimates.

The experimental data was collected using 13 optoelectronic cameras (Vicon, Oxford Metrics, UK, 100 Hz) and three ground reaction force plates embedded in the floor (AMTI, Watertown, MA, USA, 1,000 Hz). Each participant was equipped with 38 reflective markers on bony landmarks conforming to the full-body plug-in walk model (Oxford Metrics). The single markers on the body segments were substituted by rigid three marker clusters. The marker trajectories and ground reaction force data were used as input in a standard musculoskeletal modeling workflow applied in OpenSim 3.3 (Delp et al., 2007). First, the generic OpenSim model gait2392 (Delp et al., 1990) was used. We added a degree of freedom in the knee joint (i.e., ab/adduction) and implemented a functional knee axis of rotation (Meireles et al., 2017). The model was scaled to match the height and weight of the participant. Joint kinematics were derived from marker trajectories using inverse kinematics analysis with a Kalman smoothing algorithm (De Groote et al., 2008). Subsequently, joint moments were calculated with the inverse dynamic analysis using the calculated joint angles and measured ground reaction forces. Muscle force and muscles activation were determined using static optimization. Lastly, the joint contact forces were calculated using the vector sum of the estimated muscle forces and joint reaction forces (Steele et al., 2012).

Since our goal is to build a workflow that estimates the joint loading for one repetition of an exercise, we aggregate the contact forces by extracting the joint impulse *y*. This variable is defined as the integral of the contact force signal, relative to the subject's body weight:

$$y=\frac{\overset{T}{\underset{0}{\int }}CF_{t}dt}{m\cdot 9.81}$$

where *CF*_*t*_ is the joint contact force at time *t*, *T* is the duration of one exercise repetition, and *m* is the body mass. We compute the joint impulse for the left and right hip and knee.

### 2.4. Input Signals

For the input data of our joint impulse estimation models, we use inertial measurement unit (IMU) sensors since they are easy to use outside the lab. IMU sensors are often used in human motion analysis for this reason (Bussmann et al., 2001; Weyand et al., 2001; Alvarez et al., 2008; Camomilla et al., 2018). In addition, they are relatively inexpensive to buy compared to the lab equipment needed to calculate joint contact forces.

In this study, we use the IMU sensors from a mobile phone (Samsung Galaxy J5 2017). During the whole exercise protocol, the phone continuously recorded the 3D acceleration (*a*_*x*_, *a*_*y*_, *a*_*z*_) and 3D angular velocity (*g*_*x*_, *g*_*y*_, *g*_*z*_), both with a sampling rate of 50 Hz^1^. The phone was attached to the patient's left hip using a velcro strap around the patients' hips. While our goal is to predict the joint loading on both sides, we also wanted to use a simple setup with the minimal number of sensors. Hence, we only use one sensor and always attach it on the same side of the body. Since people usually wear their phone in a pocket, we chose the left hip to mimic that placement. The phone was attached such that the IMU's reference frame corresponded to the anterior-posterior (*x*), proximal-distal (*y*) and lateral-medial (*z*) direction of the person's left leg.

Since the signals change over time, each signal is represented as a time series, i.e., a sequence of values. For example, the *a*_*x*_ acceleration signal corresponds to the time series [*a*_*x*_(*t*_0_), *a*_*x*_(*t*_1_), …, *a*_*x*_(*t*_*n*_)] where *t*_*i*_ is the *i*^th^ time stamp of an exercise repetition.

### 2.5. Synchronization

Whereas the optoelectronic cameras and the ground reaction force plates were connected to the computer that was used for measuring the joint contact forces, the mobile phone sensor recordings were collected directly on the phone. Since the computer and the mobile phone recorded the data independently, their recordings were not synchronized through a single clock. In order to link to correct parts of the sensor data to the joint contact forces, both systems' time stamps have to be aligned. This can be done by incrementing the time stamps of the phone by the lag between the two clocks, i.e., the difference between the computer's clock time and the phone's clock time.

**Figure 1:**
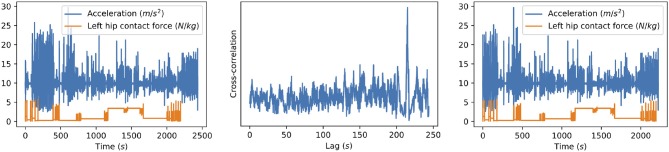
Illustration of our synchronization method for one of the subjects. The original signals **(left)** are the resultant acceleration measured by the phone and the left hip contact force. The cross-correlation between these signals **(middle)** is computed for all possible lags, i.e., all lags for which the hip contact force signal still ends before the acceleration signal ends. The location of the highest peak then corresponds to the time difference between the signals, which can be used to align the signals **(right)**.

Unfortunately, the exact lag was unknown at the time of data collection. Finding this lag manually would require to check for each possible lag whether the joint contact force signal is aligned with the phone's signals and select the lag that results in the best alignment. Additionally, since the lags vary across the different collection sessions due to drift in the phone's clock time, this would have to be repeated for each subject. Therefore, we align the signals automatically using an approach based on the cross-correlation coefficient between the signals. Specifically, we compute the cross-correlation for each possible lag, i.e., xcorr(*P*_*l*:*l*+_*n*__*CF*__, *CF*) for each lag *l*∈[0, *n*_*P*_−*n*_*CF*_], which corresponds to the lags *l* for which all time stamps of the contact force data *CF* are between the start and end of the phone data *P*. Figure 1 illustrates the synchronization approach.

### 2.6. Pipeline

**Figure 2:**
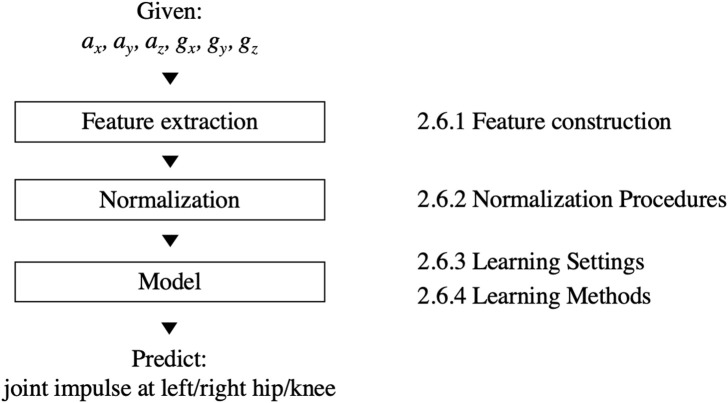
Joint loading estimation pipeline.

Figure 2 shows our machine learning pipeline for predicting the joint impulse based on the phone's signals. The input and output of the pipeline are defined as follows:

- **Input:** The measurements of the phone's IMU collected during a single exercise repetition.
- **Output:** The joint impulse at the left hip, right hip, left knee or right knee. Each location corresponds to one target variable, i.e., the goal is to predict one value per location. Since the four locations may require different models, we develop a separate pipeline (with the same building blocks) for each target.

The pipeline consists of three building blocks. First, the *feature extraction* block converts the raw phone signals into a format that is suitable for learning a model. This format consists of features that summarize the phone signals, e.g., by extracting the mean of the *a*_*x*_ signal. Each feature summarizes the data of one exercise repetition, i.e., one window of data. The process of defining a set of relevant features is called feature construction. Section 2.6.1 describes this process in more detail. Next, the *normalization* block normalizes the feature values in order to make the predictions more robust. Section 2.6.2 lists several normalization procedures. Finally, the *model* block turns the (normalized) feature values into a prediction for the left/right hip/knee joint impulse. Since the relation between phone-based features and joint impulse is unknown from a biomechanics perspective, we use machine learning to automatically learn a model from a dataset labeled with ground-truth joint impulses. Sections 2.6.3 and 2.6.4 describe the learning settings and methods for training these models.

#### 2.6.1. Feature Construction

The input of the pipeline consists of the measurements collected by the phone's sensors. However, the high dimensionality of the raw phone signals prevents using these signals directly for training a model. Therefore, we follow a feature-based approach (Fulcher, 2018) and convert the raw phone signals into a low-dimensional feature representation which captures the relevant characteristics of the signals.

We use the TSFuse Python package with the minimal feature extraction settings to generate a feature representation. This package extracts a set of statistical features (e.g., mean, median, variance,…) from both the original signals and additional signals derived from these signals. To derive new signals, TSFuse combines multiple signals using different transformations (e.g., the resultant of three signals). We refer to De Brabandere et al. (2019) for the complete list of transformations as well as the feature construction algorithm which builds features using these transformations.

Since this construction method uses the target data to remove irrelevant features, the feature construction method was repeated for each cross-validation fold (see section 2.7). In our experiments, TSFuse constructed the same 63 features in each fold. Supplementary Table 1 shows an overview of the constructed features.

#### 2.6.2. Normalization Procedures

Normalizing the feature values may be required from a machine learning and biomechanics perspective. From a machine learning perspective, standardizing the feature values to a similar range is necessary for certain types of models, including the regularized linear model of our pipeline (section 2.6.4). From a biomechanics perspective, other studies using accelerometer data have shown that individual differences (e.g., body height, body mass, movement pattern, …) may influence the signals and thus affect the feature values as well (Op De Beéck et al., 2018).

For the features, we consider the following normalization procedures:

- No normalization: use the original feature values.
- Dataset-level standardization: standardize each feature using the mean and standard deviation as computed over the complete dataset. This procedure only accounts for differences in the range of the features.
- Subject-level standardization: standardize each feature using the mean and standard deviation as computed separately for each subject. This procedure also accounts for differences between subjects.

For the target data, we only consider (1) the original joint impulses relative to subject's body weight, and (2) the standardized impulses using the mean and standard deviation over the complete training set. We do not consider standardizing based on each subject's joint impulses since that would require ground truth joint loading measurements for the test data, which is understandable as the model does not have these when applied to an unseen subject.

#### 2.6.3. Learning Settings

Since some exercises have completely different movements, the joint impulse can not be modeled in the same way for each exercise. The model could detect the exercise type itself by training the model using the complete dataset. However, given the small dataset size, we simplify the learning task by training multiple models, each focusing on only one or a few similar exercises. Specifically, we consider the following two learning settings:

- **One exercise (OE)**
  This setting splits the data per exercise type and evaluates models for each exercise type separately.
- **Similar exercises (SE)**
  This setting splits the dataset in groups of similar exercises: walk, ascend stairs and descend stairs; sit down and stand up; forward lunge and side lunge; and stand on one leg and squat on one leg.

Grouping multiple exercises in the SE setting increases the number of training examples compared to the OE setting, which may help selecting relevant features and setting good parameters for the model. We hypothesize that the SE setting yields more accurate models as a result of the increased training set size. To evaluate this hypothesis, section 3.4 compares both settings.

#### 2.6.4. Learning Methods

To estimate the joint loading based on the phone's data, we train regularized linear regression models using the Least Absolute Shrinkage and Selection Operator (LASSO) by Tibshirani (1996). This method performs both regularization and feature selection by including the ℓ_1_-norm of the weights in the cost function. Given the small dataset of this study, this method is suitable as it is able to select relevant features from a large number of features (*p*) when the number of training examples (*n*) is small (*n*≪*p*). In our experiments, we use the Lasso implementation of scikit-learn (Pedregosa et al., 2011) with the default parameters, which sets the regularization constant alpha to 1.

We compare the linear regression models to a naïve baseline model which predicts the average joint impulse of all exercise repetitions in the training data. As the baseline requires no learning, achieving a lower prediction error is a minimal requirement for the linear model to do better than the current best approach for monitoring the joint loading of patients. This approach uses the population average as a “joint loading profile” for monitoring an individual patient. The naïve baseline estimates the population average from a specific group of subjects, in this case a sample of hip OA patients.

### 2.7. Evaluation

We evaluate the pipeline's performance on unseen (i.e., future) data with respect to two scenarios: (1) applying the model to an *unseen patient*, and (2) applying the model to a *seen patient*, i.e., a patient for whom some labeled data is already available. The first scenario is relevant when a doctor without lab access applies the model to one of his patients. Since this patient's movement patterns may be different compared to the patients for whom the model was trained, we hypothesize that the second scenario may improve the predictions by including labeled data of the patient in the training data. To evaluate these scenarios, we employ the following cross-validation procedures:

- **Leave-one-subject-out cross-validation**
  This cross-validation procedure evaluates how accurate the pipeline works for an *unseen* patient. In each fold, we hold out all data of a single patient and train the model using all other patients' data. The error averaged over all folds corresponds to the prediction error that a doctor without lab access can expect.
- **Leave-one-exercise-type-out cross-validation**
  This cross-validation procedure evaluates how accurate the pipeline works for a *seen* patient. This procedure splits the data based on the exercise type. In each fold, the test data consists of all repetitions of one exercise type performed by one subject. The training data consists of all other exercises performed by the same subject as well as all data of the other subjects. Note that we do not consider leave-one-repetition-out cross-validation: since all trials of each exercise were performed consecutively, the dependency between trials may be too strong and result in overly optimistic errors.

For both cross-validation schemes, we evaluate the models by reporting the relative mean absolute error (MAE%) of the estimated joint impulses *ŷ*_*i*_ w.r.t. the ground truth joint impulses *y*_*i*_ over all exercise repetitions *i*. This metric represents the average relative deviation from the actual joint impulses over all exercise repetitions performed by a patient. The MAE% is defined as follows:

$$\text{MAE\%}=\overset{N}{\underset{i}{\sum }}|\frac{{\hat{y}}_{i}-y_{i}}{y_{i}}|$$

## 3. Results

This section evaluates the proposed joint impulse prediction pipeline. We evaluate the pipeline using both cross-validation procedures in section 3.1 (leave-one-subject-out) and section 3.2 (leave-one-exercise-type-out). For the pipeline's building blocks, we use dataset-level standardization for the feature values and train the models using the SE setting. Our comparison in sections 3.3 and 3.4 shows that this normalization procedure and learning setting were found to be optimal choices for our dataset.

### 3.1. Error for Unseen Patients

Table 2 shows the MAE% for the joint impulse at each of the four locations. Overall, the linear model outperforms the baseline for the hip joint loading. However, the knee joint loading seems harder to estimate as the linear model is marginally more accurate than the baseline for right knee and even less accurate than the baseline for the left knee. Evaluating the error for each exercise type separately shows that the results are different across different exercise types. The hip joint impulse estimations for walking show the largest improvement over the baseline compared to the other exercises.

**Table 2:** MAE% evaluated using leave-one-subject-out cross-validation.

**Exercise**	**Method**	**Left hip**	**Right hip**	**Left knee**	**Right knee**
Walk	Baseline	0.439	0.460	0.286	0.291
	Linear	**0.168**	**0.155**	0.430	0.406
Ascend stairs	Baseline	0.158	0.077	0.193	0.076
	Linear	0.158	0.077	0.193	0.183
Descend stairs	Baseline	0.184	0.340	0.207	0.164
	Linear	0.184	**0.227**	0.319	0.478
Sit down	Baseline	0.360	0.324	0.372	0.214
	Linear	0.360	0.324	**0.279**	0.214
Stand up	Baseline	0.296	0.204	0.269	0.142
	Linear	0.296	0.204	0.269	0.142
Forward lunge	Baseline	0.280	0.300	0.208	0.337
	Linear	**0.263**	**0.265**	**0.178**	**0.256**
Side lunge	Baseline	0.277	0.293	0.254	0.314
	Linear	0.325	0.330	**0.243**	**0.269**
Stand on one leg	Baseline	0.461	0.469	0.531	0.352
	Linear	0.531	**0.315**	0.744	0.401
Squat on one leg	Baseline	0.278	1.031	0.291	1.986
	Linear	**0.251**	1.081	**0.223**	**1.811**
Overall	Baseline	0.314	0.417	0.297	0.483
	Linear	**0.290**	**0.360**	0.321	**0.482**

*The errors which outperform the baseline are highlighted in bold*.

### 3.2. Error for Seen Patients

Table 3 compares the leave-one-subject-out cross-validation scheme with the leave-one-exercise-type-out cross-validation. We hypothesized that the leave-one-exercise-type-out cross-validation could improve the predictions by including data of the patient in the test data. Unfortunately, the leave-one-exercise-type-out errors are close to the leave-one-subject-out errors and for the left and right hip, the leave-one-subject-out models often outperform the leave-one-exercise-type-out models.

**Table 3:** MAE% for the two cross-validation schemes.

	**Left hip**	**Right hip**	**Left knee**	**Right knee**
Leave-one-subject-out	**0.290**	**0.360**	0.321	0.482
Leave-one-exercise-type-out	0.296	0.407	**0.295**	0.482

*For each location, the lowest error is highlighted in bold, if one cross-validation method outperforms the other*.

### 3.3. Comparison of Normalization Procedures

In section 2.6.2, we hypothesized that normalization procedures can improve the error of the models by scaling features to a similar range and removing inter-individual differences. Table 4 compares all possible combinations of the normalization procedures for both the features and the target data by reporting the overall MAE% averaged over all locations (left and right hip and knee) for each of combination. The best performing combination is the dataset-level feature standardization and no target normalization. Surprisingly, subject-level standardization does not result in more accurate models compared to dataset-level standardization.

**Table 4:** Overall MAE% (averaged over all locations, i.e., left/right hip/knee) for different combinations of the normalization procedures.

		**Target normalization**	
		No	Yes
Feature normalization	No	0.439	0.433
	Dataset-level	**0.363**	0.391
	Subject-level	0.371	0.391

*The table shows the results for the linear models with the SE setting evaluated using leave-one-subject-out cross-validation. The lowest error over all combinations is highlighted in bold*.

### 3.4. Comparison of Learning Settings

In section 2.6.3, we hypothesized that the SE setting yields more accurate results as this setting increases the number of training examples by combining multiple exercises. To evaluate this hypothesis, Table 5 compares the SE setting with the OE setting. Overall, the SE models are more accurate than the OE models for all locations except for the left knee. For the hip joint impulse, the SE models show the largest improvement for walking. However, the results are slightly less accurate for other exercises (e.g., forward lunge and side lunge) which indicates that the SE models are suitable for walking but not for other exercises.

**Table 5:** MAE% of the similar exercises (SE) models and one exercise (OE) models.

**Exercise**	**Setting**	**Left hip**	**Right hip**	**Left knee**	**Right knee**
Gait	OE	0.439	0.460	0.286	0.291
	SE	**0.168**	**0.155**	0.430	0.406
Ascend stairs	OE	0.158	0.077	0.193	0.076
	SE	0.158	0.077	0.193	0.183
Descend stairs	OE	0.184	0.340	0.207	0.164
	SE	0.184	**0.227**	0.319	0.478
Sit down	OE	0.360	0.324	0.380	0.214
	SE	0.360	0.324	**0.279**	0.214
Stand up	OE	0.296	0.204	0.269	0.142
	SE	0.296	0.204	0.269	0.142
Forward lunge	OE	0.202	0.265	0.174	0.264
	SE	0.263	0.265	0.178	**0.256**
Side lunge	OE	0.277	0.293	0.254	0.314
	SE	0.325	0.330	**0.243**	**0.269**
Stand on one leg	OE	0.462	0.292	0.582	0.352
	SE	0.531	0.315	0.744	0.401
Squat on one leg	OE	0.196	1.167	0.242	2.058
	SE	0.251	**1.081**	**0.223**	**1.811**
Overall	OE	0.296	0.407	0.295	0.482
	SE	**0.290**	**0.360**	0.321	0.482

*The SE errors which outperform the OE errors are highlighted in bold*.

## 4. Discussion

The goal of this study was to explore the possibility of using a minimal number of sensors for predicting joint loading in hip OA patients. We proposed a machine learning pipeline that requires only the IMU data collected from a mobile phone. In this section, we discuss our choices for the different building blocks of this pipeline. We then discuss the differences in the obtained errors with respect to the joints and exercise types. Finally, we discuss the accuracy vs. ease-of-use trade-off of our approach and suggest future directions with respect to this trade-off.

### 4.1. Building Blocks of the Machine Learning Pipeline

The proposed machine learning pipeline required making several decisions for the different building blocks. For the *feature extraction* block, we used an automated approach (De Brabandere et al., 2019) to define the feature representation. For the *normalization* block, we compared different normalization procedures and found that dataset-level feature standardization was important. For the *model* block, we only considered a linear regression model, since the small dataset size prevented us from using non-linear models. Whereas we used the pipeline for predicting the joint impulses of the hip and knee, it could be relevant for other locations as well. It could also be interesting to explore whether this pipeline (potentially with a non-linear model) can be used for other types of exercises and for other types of sensors as input.

### 4.2. Errors Across Different Joints

The results of Table 2 show a clear difference in accuracy for the hip and knee joints. The obtained results indicate that the proposed pipeline is able to predict the hip impulse, but it remains hard to outperform the naïve baseline for the knee impulse. Perhaps placing the IMU closer to the target joint might lead to better results in predicting knee contact forces. An IMU sensor on the hip might not capture the higher linear accelerations and angular velocities that are found on the segments connected to the knee joint. Considering the body's ability to attenuate shock, the acceleration signal amplitude has already weakened when reaching the IMU placed at hip level (Kavanagh and Menz, 2008). Placing an IMU on the shank could better capture these initial loading shocks (distal part of the shank), or higher acceleration signals (middle part of the shank). However, which placement is best to obtain better joint loading predictions should be investigated. Therefore, different IMU placements should be investigated to examine if personalizing the placement based on the type of patient (i.e., hip or knee osteoarthritis patient) leads to better joint loading prediction results during these types of exercises.

Similar to the difference between the hip and knee, there are also different errors for the left and right side. Interestingly, this difference does not only hold for the linear models, but also for the baselines, which suggests that there may be a larger variability in the joint impulses for the right side compared to the left side. One possible reason could be the side of the hip that was affected. However, this is unlikely as the right hip was affected for 6 patients and the left hip for 4 patients. To evaluate whether the difference between left and right is significant, we performed a two-sided paired t-test for the overall MAE% of the baseline. That is, we tested whether [*x*_1_, …, *x*_10_] is significantly different from [*y*_1_, …, *y*_10_] where *x*_*i*_ is subject *i*'s MAE% of the baseline for the left hip/knee and *y*_*i*_ is subject *i*'s MAE% of the baseline for the right hip/knee. The resulting p-values are 0.3694 (for the hip) and 0.4458 (for the knee) meaning that the difference between left and right was not significant. Most likely, the difference is due to the small sample size (only 10 subjects) and does not hold in general.

### 4.3. Errors Across Different Exercise Types

**Figure 3:**
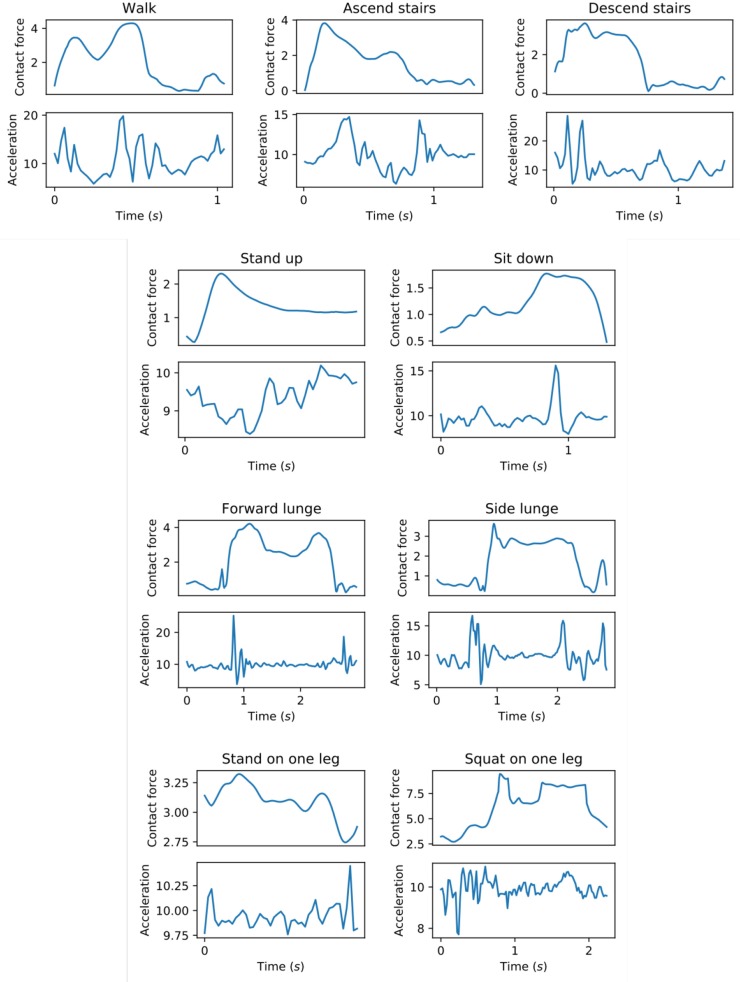
Hip contact force (*N*/*kg*) along with the resultant acceleration (*m*/*s*^2^) as measured by the mobile phone. For each exercise type, the figure shows a single repetition performed by one subject.

The errors of the linear model are different for the different exercise types, which suggests that predicting joint impulses is easier for some exercises compared to others. Given that there are large differences in the movements between exercises, the differences in the joint impulse prediction errors can depend on the relation between the data collected by the phone and the contact force at each point in time. Figure 3 shows these contact forces along with the resultant acceleration for each exercise type. For those exercises for which both the left and right hip joint impulse predictions are better than the baseline (walk and forward lunge), the contact force signal shows two main peaks for which also the resultant acceleration has a clear peak. The potential relation between the height of these acceleration peaks and the height of the contact force peaks could make the prediction of the joint impulses easier.

### 4.4. Selected Features

The linear models in the *model* block were trained using L1-regularization (lasso), which select a small number of features out of the 63 generated features. Since the selected features are different for each fold, it is hard to visualize which features are used in the models given the large number of models (10 subjects, 4 groups of exercises and 4 locations result in 160 models). Instead, we run stability selection (Meinshausen and Bühlmann, 2010) for each group of exercises and for each location to get an idea of which features were selected most often. Stability selection repeatedly trains Lasso models (with random subsampling) and reports each feature's importance as the percentage of models in which the feature was used. Supplementary Table 2 shows the top 5 features with the corresponding importance scores, for each location and each group of exercises. One observation is that sum and length features are commonly used. Since the joint impulse is defined as an integral of the contact force, it is expected that this feature is important to capture the duration of the exercise repetition. Unfortunately, it is hard to interpret the importance of the other selected features. Future work could explore using more specific (manually handcrafted) features when the goal is to get a better insight in the learned models.

### 4.5. Trade-Off Between Accuracy and Ease-of-Use

This study explores a trade-off between accuracy and ease-of-use. The most accurate model would be the one that uses all lab equipment needed for calculating joint contact forces using a musculoskeletal modeling workflow. However, this model would also be the most inconvenient as it requires the patient to come to the lab (which is probably not located in the hospital), attach 38 reflective markers to the patient and analyze the collected data in order to calculate the joint contact forces from the collected measurements.

Our model only requires attaching a mobile phone to the patient's left hip^2^. Given that a patient consultation typically takes approximately only 15 min, using a small number of sensors is an important requirement for developing a joint loading estimation tool. In addition, using a mobile phone reduces the cost of such a tool, since clinicians most likely already own a mobile phone and only need to install an app to apply the model.

However, given the results of this work, we recognize that using a mobile phone may be an easy solution, but unfortunately, one that is not accurate enough for valid clinical use. A better compromise between accuracy and ease-of-use would be to use a combination of IMU sensors at more than one location. This would allow having a better view of the patient's movements. Still, the number of sensors should be kept to a minimum in order to keep the tool practical. More research is needed to evaluate which locations are most suitable.

Even though the results are far from perfect, we argue that our phone-based model is a step in the right direction in estimating joint loading in a clinical setting using a very limited amount of sensors. Especially the results for predicting the joint impulse during level walking are interesting, where distinct reduction in mean absolute error from the baseline can be seen (MAE% from 43.9 to 16.8%). When monitoring a patient during daily life, this result is promising as walking is one of the most commonly performed daily activities and might be responsible for the majority of the joint loading during a day. The improvement over the baseline indicates that clinicians are able to obtain more accurate estimates of a patient's joint load compared to using a population average. In the future, a “hip OA” profile using population averages could shift to a “personal” profile using a more individualized estimate of joint loading. This in turn could help clinicians align a person's exercise prescription to their individual loading profile based on more accurate methods which could improve their rehabilitation. Given that joint contact forces are believed to be important in the initiation and progression of OA (Felson, 2013), this might be a promising tool in the rehabilitation setting to asses patients' joint impulses during walking over time and adjust the rehabilitation and exercise prescription accordingly.

## 5. Conclusion and Future Work

This work presented a machine learning pipeline to estimate the hip and knee joint impulse based on a mobile phone. In terms of the mean absolute error, we found that the proposed pipeline is able to slightly outperform a population average baseline for the hip (left hip: 29.0% for the linear model vs. 31.4% for the baseline; left hip: 36.0 vs. 41.7% for the right hip), but not for the knee. Our approach has two key advantages over existing methods for predicting joint loading. First, the proposed pipeline only requires a mobile phone as input. Second, we trained and evaluated the pipeline using data of patients instead of healthy subjects, which is relevant with respect to the setting in which the proposed pipeline is applicable, i.e., monitoring patients.

However, even though our phone-based model is a step in the direction of estimating joint contact forces using a minimal number of sensors, the current approach still has several limitations that need to be addressed in future work. First, the overall error of our approach should be reduced further in order to be applicable in a clinical context. One possibility is to use multiple sensors, but still only a few such that the model remains easy to use. Related work by Wesseling et al. (2018) has shown that a combination of six IMU kinematic variables can estimate hip joint loading but that for accurate knee joint loading estimates both kinematic variables and ground reaction forces are needed. Future work can investigate how to extract sufficiently informative features from a minimal number of sensors. For example, extracting joint angles could improve the prediction error (McLean et al., 2003), but this requires at least two sensors. Second, while we always attached the phone at a fixed position, the phone's orientation could be slightly different due to variations across experiments with different subjects. This means that our learned models are evaluated on data that may have been collected using a slightly different reference frame for the sensor measurements. Hence, our model should be robust against minor perturbations of the phone's orientation, but not against attaching the phone at different locations. Future work should develop models that are robust against variations in the position of the sensors as well. This can be done by collecting data with sensors at different locations and using machine learning to train a model that works for various locations. Third, we decided to only use linear models, since non-linear models did not improve the results for this small dataset of 10 patients. Training non-linear models using data from more patients can potentially detect non-linear relations between the sensor data and the joint impulse. Moreover, additional data can improve the model's accuracy by learning from a larger number of training examples.

## Data Availability Statement

The datasets generated for this study are available on request to the corresponding author.

## Ethics Statement

The studies involving human participants were reviewed and approved by Ethics Committee Research UZ/KU Leuven. The patients/participants provided their written informed consent to participate in this study.

## Author Contributions

AD, JE, AT, IJ, BV, and JD conceived, designed and coordinated the study. JE collected the data. AD and JD developed the machine learning pipeline. AD and JE analyzed the data and initially drafted the manuscript. AT, IJ, BV, and JD provided useful suggestions in the preparation of the final manuscript.

### Conflict of Interest

The authors declare that the research was conducted in the absence of any commercial or financial relationships that could be construed as a potential conflict of interest.

## Footnotes

^1^We recorded the data from the TYPE_ACCELEROMETER and TYPE_GYROSCOPE sensor types of Android. As shown in the API documentation (https://developer.android.com/guide/topics/sensors/sensors_motion). Android corrects for drift in the sensor measurements.

^2^Alternatively, the patient could wear the phone in his left pocket, but further research is needed to evaluate whether this does not decrease the accuracy of the estimations. Both ways of wearing the phone are convenient in a clinician's practice.
