# Supplementary material for: A Machine Learning Approach to Estimate Hip and Knee Joint Loading Using a Mobile Phone-Embedded IMU
Source: Front Bioeng Biotechnol. 2020 Apr 15;8:320. doi: 10.3389/fbioe.2020.00320 (PMC7174587; doi:10.3389/fbioe.2020.00320)
Supplement: Supplementary file 1 [file Data_Sheet_1.PDF]

Supplementary Table 1. Overview of all 63 features generated using TSFuse.

Accelerometer features

Length(a_x)
Length(a_y)
Length(a_z)
Sum(a_x)
Sum(a_y)
Sum(a_z)
Min(a_x)
Min(a_y)
Min(a_z)
Max(a_x)
Max(a_y)
Max(a_z)
Mean(a_x)
Mean(a_y)
Mean(a_z)
Median(a_x)
Median(a_y)
Median(a_z)
Variance(a_x)
Variance(a_y)
Variance(a_z)
StandardDeviation(a_x)
StandardDeviation(a_y)
StandardDeviation(a_z)
Skewness(a_x)
Skewness(a_y)
Skewness(a_z)
Kurtosis(a_x)
Kurtosis(a_y)
Kurtosis(a_z)
Length(Resultant(a_x, a_y, a_z))
Sum(Resultant(a_x, a_y, a_z))
Median(Resultant(a_x, a_y, a_z))

Gyroscope features

Length(g_x)
Length(g_y)
Length(g_z)
Sum(g_x)
Sum(g_y)
Sum(g_z)
Min(g_x)
Min(g_y)
Min(g_z)
Max(g_x)
Max(g_y)
Max(g_z)
Mean(g_x)
Mean(g_y)
Mean(g_z)
Variance(g_x)
Variance(g_y)
Variance(g_z)
StandardDeviation(g_x)
StandardDeviation(g_y)
StandardDeviation(g_z)
Skewness(g_x)
Skewness(g_y)
Skewness(g_z)
Kurtosis(g_x)
Kurtosis(g_y)
Kurtosis(g_z)
Length(Resultant(g_x, g_y, g_z))
Sum(Resultant(g_x, g_y, g_z))
Median(Resultant(g_x, g_y, g_z))
