# Supplementary material for: A Machine Learning Approach to Estimate Hip and Knee Joint Loading Using a Mobile Phone-Embedded IMU
Source: Front Bioeng Biotechnol. 2020 Apr 15;8:320. doi: 10.3389/fbioe.2020.00320 (PMC7174587; doi:10.3389/fbioe.2020.00320)
Supplement: Supplementary file 2 [file Data_Sheet_2.pdf]

Supplementary Table 2. Feature importances calculated using stability selection, for each group of exercises and for each location. Stability selection repeatedly trains Lasso models (with random subsampling) and reports each feature's importance as the percentage of models in which the feature was selected. The table shows the top-5 features with the corresponding importance scores.

	Walking + ascend stairs + descend stairs		Stand up + sit down		Forward lunge + side lunge		Stand on one leg + squat on one leg	
	Feature	*Score*	*Feature*	*Score*	*Feature*	*Score*	*Feature*	*Score*
**Left hip**	Mean(a_z)	0.5880	Min(a_z)	0.5125	Median(Resultant(a_x, a_y, a_z))	0.8405	Sum(a_z)	0.5765
	Max(a_x)	0.4680	Sum(Resultant(a_x, a_y, a_z))	0.4550	Skewness(g_z)	0.7860	Sum(a_x)	0.5025
	Sum(Resultant(a_x, a_y, a_z))	0.4045	Skewness(a_y)	0.3475	Skewness(g_x)	0.6845	Sum(Resultant(a_x, a_y, a_z))	0.4610
	Skewness(a_y)	0.3660	Sum(g_y)	0.3235	Sum(a_z)	0.6560	Max(g_x)	0.4320
	Length(a_x)	0.3370	Mean(a_z)	0.2340	Min(a_x)	0.6045	Variance(g_x)	0.3950
**Right hip**	StandardDeviation(a_y)	0.4955	Kurtosis(g_y)	0.7690	Skewness(a_y)	0.7465	Sum(a_x)	0.7345
	Sum(Resultant(a_x, a_y, a_z))	0.3670	Skewness(g_z)	0.6595	Sum(a_x)	0.5400	Skewness(g_x)	0.6435
	StandardDeviation(a_x)	0.2610	Kurtosis(g_x)	0.6070	Max(a_x)	0.4855	Sum(a_z)	0.5885
	Kurtosis(g_y)	0.2350	Sum(a_z)	0.5395	Sum(Resultant(a_x, a_y, a_z))	0.4545	Kurtosis(g_z)	0.4750
	Variance(a_y)	0.2240	Sum(a_y)	0.5175	StandardDeviation(a_y)	0.3855	Max(g_x)	0.2995
**Left knee**	Length(a_x)	0.4070	StandardDeviation(g_x)	0.4540	Median(Resultant(a_x, a_y, a_z))	0.7570	Sum(a_z)	0.5135
	Sum(Resultant(a_x, a_y, a_z))	0.3485	Min(a_z)	0.4410	Skewness(g_x)	0.6880	StandardDeviation(a_z)	0.4700
	Skewness(g_y)	0.3305	Kurtosis(a_x)	0.4055	Sum(Resultant(a_x, a_y, a_z))	0.4695	Sum(Resultant(a_x, a_y, a_z))	0.4640
	Skewness(a_y)	0.3090	Sum(Resultant(a_x, a_y, a_z))	0.3760	Skewness(g_z)	0.4605	Sum(Resultant(g_x, g_y, g_z))	0.4605
	Length(a_y)	0.2600	Variance(a_y)	0.2990	Sum(a_x)	0.4040	Variance(a_y)	0.4530
**Right knee**	Kurtosis(g_y)	0.5065	Skewness(g_z)	0.5540	Skewness(a_y)	0.7135	Sum(a_x)	0.8020
	Sum(Resultant(a_x, a_y, a_z))	0.4770	StandardDeviation(a_y)	0.5540	Median(Resultant(a_x, a_y, a_z))	0.5140	Median(Resultant(a_x, a_y, a_z))	0.7880
	Skewness(g_y)	0.4735	Kurtosis(a_y)	0.4980	Sum(a_x)	0.5065	Skewness(g_x)	0.6410
	Kurtosis(g_z)	0.4655	StandardDeviation(g_z)	0.4760	Sum(a_y)	0.4875	Sum(a_z)	0.6105
	Kurtosis(a_x)	0.3950	Sum(a_y)	0.4590	Sum(Resultant(a_x, a_y, a_z))	0.4560	Sum(Resultant(g_x, g_y, g_z))	0.5995
